# A meta-analysis of sublingual allergen immunotherapy and pharmacotherapy in pollen-induced seasonal allergic rhinoconjunctivitis

**DOI:** 10.1186/1741-7015-12-71

**Published:** 2014-05-01

**Authors:** Philippe Devillier, Jean-François Dreyfus, Pascal Demoly, Moisés A Calderón

**Affiliations:** 1 UPRES EA 220 & Clinical Research Department, Foch Hospital, University of Versailles Saint-Quentin, Suresnes, France; 2 Biostatistics Unit, Clinical Research Department, Foch Hospital, Suresnes, France; 3 EPAR INSERM U707, Allergy Division, Pulmonology Department, Hôpital Arnaud de Villeneuve, University Hospital of Montpellier, Montpellier, France, and Institut Pierre Louis d’Epidémiologie et de Santé Publique, Faculté de Médecine, Université Pierre et Marie Curie, Paris, France; 4 Section of Allergy and Clinical Immunology, Imperial College London-NHLI, Royal Brompton Hospital, Dovehouse Street, London, UK

**Keywords:** Allergen immunotherapy, Rhinoconjunctivitis, Grass, Pollen, Effect size, Pharmacotherapy

## Abstract

**Background:**

The capacity of sublingual allergen immunotherapy (SLIT) to provide effective symptom relief in pollen-induced seasonal allergic rhinitis is often questioned, despite evidence of clinical efficacy from meta-analyses and well-powered, double-blind, placebo-controlled randomized clinical trials. In the absence of direct, head-to-head, comparative trials of SLIT and symptomatic medication, only indirect comparisons are possible.

**Methods:**

We performed a meta-analysis of classes of products (second-generation H1-antihistamines, nasal corticosteroids and grass pollen SLIT tablet formulations) and single products (the azelastine-fluticasone combination MP29-02, and the leukotriene receptor antagonist montelukast) for the treatment of seasonal allergic rhinitis in adults, adolescents and/or children. We searched the literature for large (n >100 in the smallest treatment arm) double-blind, placebo-controlled randomized clinical trials. For each drug or drug class, we performed a meta-analysis of the effect on symptom scores. For each selected trial, we calculated the relative clinical impact (according to a previously published method) on the basis of the reported post-treatment or season-long nasal or total symptom scores: 100 × (score_Placebo_ - score_Active_)/score_Placebo_.

**Results:**

Twenty-eight publications on symptomatic medication trials and ten on SLIT trials met our selection criteria (total number of patients: n = 21,223). The Hedges' g values from the meta-analyses confirmed the presence of a treatment effect for all drug classes. In an indirect comparison, the weighted mean (range) relative clinical impacts were -29.6% (-23% to -37%) for five-grass pollen SLIT tablets, -19.2% (-6% to -29%) for timothy pollen SLIT tablets, -23.5% (-7% to -54%) for nasal corticosteroids, -17.1% (-15% to -20%) for MP29-02, -15.0% (-3% to -26%) for H1-antihistamines and -6.5% (-3% to -10%) for montelukast.

**Conclusions:**

In an indirect comparison, grass pollen SLIT tablets had a greater mean relative clinical impact than second-generation antihistamines and montelukast and much the same mean relative clinical impact as nasal corticosteroids. This result was obtained despite the presence of methodological factors that mask the clinical efficacy of SLIT for the treatment of seasonal allergic rhinitis.

## Background

Allergic rhinitis (AR) is one of the most common chronic conditions worldwide [1-4]. Its high prevalence creates a significant medical burden through sleep disorders, mood disorders and impaired social functioning and performance at work [5-9]. This medical burden is associated with a significant economic burden (estimated at $3.4 billion in direct costs per year in the United States) [10].

The treatment goal in AR is to provide clinically relevant symptom relief and improve the patient's quality of life. Current international and national guidelines broadly agree on the therapeutic approach [1-4,11-13]. As a front-line treatment, H1-antihistamines are indicated in cases of mild or intermittent respiratory allergy and can be combined with nasal corticosteroids if the symptoms are not sufficiently relieved. Allergen immunotherapy (AIT) is a guideline-recommended therapeutic option for seasonal allergic rhinitis (SAR) [1-4]. It can be administered as subcutaneous allergen immunotherapy (SCIT) or sublingual allergen immunotherapy (SLIT); SLIT is considered to have a better safety profile than SCIT, since most adverse events are local and transient and do not lead to interruption or cessation of treatment [14,15]. Large-scale, double-blind, placebo-controlled (DBPC) randomized clinical trials, position papers and meta-analyses have emphasized the efficacy and safety of SLIT [16-22]. Drop and tablet formulations of grass pollen SLIT products have been approved by regulatory agencies in many countries for the treatment of seasonal allergic rhinoconjunctivitis in adults and in children over the age of five. However, variations in study design, patient selection, efficacy endpoints, allergen formulation, product standardization and other parameters may have given some physicians the impression that AIT products (whether SCIT or SLIT) do not have a great impact on symptoms. Direct, head-to-head comparison of AIT with symptomatic medication is methodologically complicated, not least because patients in AIT clinical trials are allowed to take symptomatic ‘rescue’ medications when they wish. Hence, only indirect comparisons are currently feasible. Matricardi *et al*. compared SCIT with symptomatic medications by calculating the relative clinical impact (RCI) [23]. The RCI is defined as the percentage difference between the total symptom score (TSS) or total nasal symptom score (TNSS) obtained for active treatment versus that obtained for placebo (see the Methods section and [23]). When considering TSSs, Matricardi *et al*. concluded that the weighted mean RCI of SCIT (-32.9 ± 12.7%) was significantly greater than that of the antihistamine desloratadine (-12.0 ± 5.1%). Similarly, when considering TNSSs, the weighted mean RCI for SCIT (-34.7 ± 6.8%) was significantly greater than that of the corticosteroid mometasone (-31.7 ± 16.7%) and the leukotriene receptor antagonist montelukast (-6.3 ± 3.0%) [23].

Matricardi *et al*. reported on SCIT but not SLIT. Hence, we decided to indirectly compare the RCIs of tablet formulations of SLIT with the values for pharmacotherapy (oral second-generation H1-antihistamines, nasal corticosteroids, the combined azelastine-fluticasone nasal spray MP29-02 and the leukotriene receptor antagonist montelukast) in exactly the same manner. We considered recent, well-powered, DBPC, randomized clinical trials in SAR.

## Methods

### Study and data selection

We searched the literature for well-powered, double-blind, randomized, controlled trials evaluating SLIT tablets, H1-antihistamines, nasal corticosteroids, an azelastine-fluticasone combination or leukotriene receptor antagonists having been granted marketing authorization within the last 16 years (that is, 1997 to 2013) for the indication of grass, tree or ragweed pollen-induced SAR in adults and/or children. MEDLINE, Embase and the Cochrane Library were searched using logical combinations of the following terms: rhiniti*; allerg*; seasonal*; rhinoconj*; hay fever; immunotherap*; immunolog*; desensiti*; grass*; pollen*; pollinos*; SAR.

When performing meta-analyses, study selection is of the utmost importance. We excluded trials with fewer than 100 participants in the placebo arm or the active treatment arm, trials lacking a true placebo group, challenge chamber studies and meta-analyses. The threshold of 100 participants per arm was considered to be justified, since it (1) enabled the selection of all the SLIT tablet studies and the vast majority of the pharmacotherapy studies and (2) prevented the selection of underpowered studies. In fact, underpowered studies often suffer from publication bias and contribute little information to meta-analyses when two or more adequately powered large trials are available [24-26]. We found at least three large studies for each single drug or drug class, whereas most of the remaining studies were small. Hence, we included only well-powered, large, multicenter, DBPC randomized clinical trials of symptomatic treatments and grass pollen SLIT tablets at the registered doses. In a recent report by Di Bona *et al*. [22], a subgroup analysis according to the number of centers showed that efficacy was higher in small single-center studies than in multicenter studies. This difference could be due to (1) publication bias and (2) exposure to more homogeneous environmental conditions in single center studies. In turn, this would lead to less variability in the treatment response and a subsequently greater effect size (relative to a multicenter study in which subjects from different regions or even different countries are enrolled). In order to increase consistency, reduce heterogeneity and compensate for this bias, we selected multicenter studies of symptomatic medications with at least 100 patients in one arm (all the SLIT tablet studies were large, multicenter studies).

Identified articles were cross-checked against those listed in recent meta-analyses and reviews.

We extracted the following data from each selected publication: (1) the active treatment and the dose, (2) the number of participants in the full analysis set or the intention-to-treat population in each treatment arm, (3) the treatment duration (or, if several treatment endpoints were quoted, the duration corresponding to the subsequently calculated RCI), (4) the nature of the symptom score used (a TNSS, a total ocular symptom score (TOSS) and/or the rhinoconjunctivitis total symptom score (RTSS)) and the number of symptoms scored. Indirect symptom scores involving predominantly sleep-related parameters (difficulty going to sleep, night-time awakenings and so on) were not analyzed. Twelve- or 24-hour reflective scores were selected in all cases.

In the SLIT tablet trials, the most frequently used criterion for symptom severity (and, thus, for calculation of the RCI in the present study) was the mean daily RTSS over the whole pollen period. The RTSS comprises four nasal symptoms and two ocular symptoms, each of which is scored on a 4-point scale from 0 (the absence of symptoms) to 3 (severe symptoms). In some trials, various symptom-medication or adjusted scores were also evaluated, such as (1) a mean symptom-medication score (RTSS/6 + (rescue medication score)/2), ranging from 0 to 3), (2) the daily average adjusted symptom score (AAdSS, in which a last-observation-carried-forward method is used to adjust the daily RTSS for rescue medication use [27]) and (3) the total combined score, which is the sum of the daily RTSS and the daily medication score [16,17].

### Meta-analysis and calculation of the RCI

Symptom scores and (for SLIT only) combined symptom-medication scores were assessed as outcome measures of the treatment effect. Hedges' g was used to express mean difference effect sizes. The I^2^ statistic was used to quantify heterogeneity and reported *P* values are based on the Q statistic (a statistic used for multiple significance testing across a number of means). Meta-analyses were performed for each drug class (or, for montelukast and MP29-02, for each single drug). When the studies’ results differed only by the sampling error (that is, no heterogeneity), a fixed-effects model was applied to estimate the overall Hedges' g using the MIX Pro add-on to Excel (version 2.0.1.4., [28]). The I^2^ statistic can be interpreted as the percentage of the total variability in a set of effect sizes due to true heterogeneity (that is, between-study variability). We considered that I^2^ values above 50% corresponded to substantial heterogeneity. When substantial heterogeneity was observed, we performed a sensitivity analysis by pooling data in a random effects model and comparing the result with that of a fixed effects model.

Given that effect sizes based on mean differences (whether Hedges' g or the standardized mean difference) do not measure the efficacy classically measured in clinical trials, we used the mean post-treatment or seasonally averaged symptom scores in the active treatment and placebo groups to calculate the RCI. We analyzed each class of symptomatic medication (or single medication for MP29-02 and montelukast) and SLIT tablets in SAR by calculating the RCI as 100 × (score_Placebo_ - score_Active_)/score_Placebo_) [23,29]. The RCIs were compared in a Kruskal-Wallis test with correction for ties. Individual comparisons were performed after correction with Simes' improved Bonferroni procedure. For the SLIT tablet studies, we also calculated the weighted mean RCI on the basis of the combined symptom-medication scores.

## Results

### Study selection

**Figure 1:**
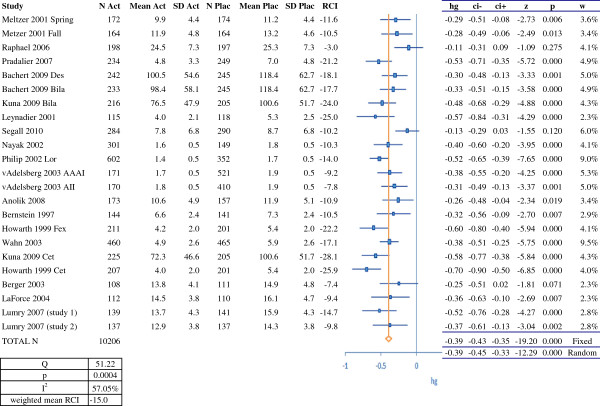
**RCI and meta-analysis of efficacy for H1-antihistamines (on the basis of symptom scores).** N Act: number of subjects in the active treatment group; Mean Act: mean score in the active treatment group; SD Act; standard deviation for the score in the active treatment group; N Plac: number of subjects in the placebo group; Mean Plac: mean score in the placebo group; SD Plac: standard deviation for the score in the placebo group; RCI: relative clinical impact; hg: Hedges' g; ci-: lower confidence interval; ci+: upper confidence interval; z: z score: p: *p*-value; w: weighting; Des: desloratadine; Bila: bilastine; Lor: loratadine; Fex: fexofenadine; Cet: cetirizine. RCI, relative clinical impact.

**Figure 2:**
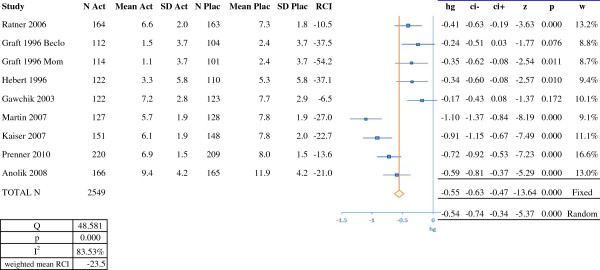
**RCI and meta-analysis of efficacy for nasal corticosteroids (on the basis of symptom scores).** N Act: number of subjects in the active treatment group; Mean Act: mean score in the active treatment group; SD Act; standard deviation for the score in the active treatment group; N Plac: number of subjects in the placebo group; Mean Plac: mean score in the placebo group; SD Plac: standard deviation for the score in the placebo group; RCI: relative clinical impact; hg: Hedges' g; ci-: lower confidence interval; ci+: upper confidence interval; z: z score: p: *p*-value; w: weighting; Beclo: beclomethasone. Mom: mometasone. RCI, relative clinical impact.

**Figure 3:**
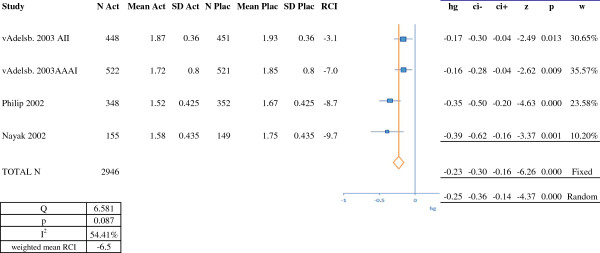
**RCI and meta-analysis of efficacy for montelukast (on the basis of symptom scores).** N Act: number of subjects in the active treatment group; Mean Act: mean score in the active treatment group; SD Act; standard deviation for the score in the active treatment group; N Plac: number of subjects in the placebo group; Mean Plac: mean score in the placebo group; SD Plac: standard deviation for the score in the placebo group; RCI: relative clinical impact; hg: Hedges' g; ci-: lower confidence interval; ci+: upper confidence interval; z: z score: p: *p*-value; w: weighting. RCI, relative clinical impact.

**Figure 4:**
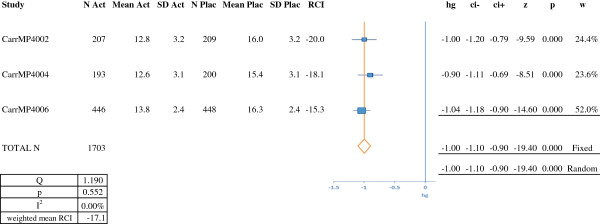
**RCI and meta-analysis of efficacy for an azelastine-fluticasone combination (on the basis of symptom scores).** N Act: number of subjects in the active treatment group; Mean Act: mean score in the active treatment group; SD Act; standard deviation for the score in the active treatment group; N Plac: number of subjects in the placebo group; Mean Plac: mean score in the placebo group; SD Plac: standard deviation for the score in the placebo group; RCI: relative clinical impact; hg: Hedges' g; ci-: lower confidence interval; ci+: upper confidence interval; z: z score: p: *p*-value; w: weighting. RCI, relative clinical impact.

For symptomatic medications, a total of 50 studies were initially selected. Twenty-two of these studies were then excluded because they failed to report (or did not enable calculation of) post-treatment scores, which prevented calculation of the RCI [see Additional file 1: Table S1] [23,29]. Hence, 28 publications on symptomatic medication trials met our selection criteria [30-57] and were analyzed further (Figures 1, 2, 3 and 4 and Additional file 2: Table S2). One publication reported on both spring and fall pollen periods [30] and several publications reported on different medications in separate treatment arms or several different trials of the same medication [34,37-40,43,57], meaning that we took account of a total of 39 separate active treatment arms (23 for antihistamines, 9 for nasal corticosteroids, 4 for montelukast and 3 for MP29-02 (a novel nasal spray formulation of azelastine and fluticasone propionate)). The selected symptomatic medication trials had been performed in study populations comprising a mixture of children, adolescents and adults, with the exception of four studies in adults only (18 or more years old) [33,36,37,51] and one study in children only (6 to 11 years old) [46]. All but seven of the trials had been performed in the United States. Symptoms were almost always rated on a 4-point scale (0 = absent; 1 = mild; 2 = moderate; 3 = severe). The most frequent symptom score was an eight-symptom TSS (T8SS) for antihistamine trials and a four-symptom TNSS (T4NSS: rhinorrhea, nasal congestion, sneezing and nasal itching) for nasal corticosteroids and montelukast. Three studies reported a TNSS and a TOSS separately [53-55].

**Figure 5:**
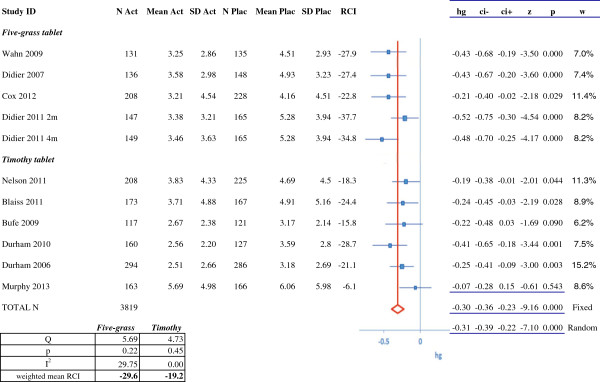
**RCI and meta-analysis of efficacy (symptom scores) for five-grass pollen SLIT tablets and timothy pollen SLIT tablets.** N Act: number of subjects in the active treatment group; Mean Act: mean score in the active treatment group; SD Act; standard deviation for the score in the active treatment group; N Plac: number of subjects in the placebo group; Mean Plac: mean score in the placebo group; SD Plac: standard deviation for the score in the placebo group; RCI: relative clinical impact; hg: Hedges' g; ci-: lower confidence interval; ci+: upper confidence interval; z: z score: p: *p*-value; w: weighting. RCI, relative clinical impact.

Eleven publications on SLIT tablet trials met our selection criteria [16-19,58-64] (Figure 5 and Additional file 2: Table S2). Three of the eleven SLIT tablet trials had been performed in children and adolescents (5 to 17 years old) [17,60,61], with the remainder in adults only (that is, patients aged 18 or over). Four and seven of the trials had been performed in the United States and Europe, respectively. Although two of the SLIT trials tested different dosages of allergen [58,59], only the data for the subsequently registered dosage (300 index of reactivity (IR) and 75,000 standardized quality tablet (SQ-T) units) were considered in the present study. The 11 SLIT tablet studies were further subdivided into those testing five-grass pollen extracts [19,59,60,62,64] and those testing *Phleum pretense* (timothy) pollen extracts [16-18,58,61,63]. For the purposes of our analysis, we studied data from single-season studies or from the last year of treatment in multiple-season studies. The study by Horak *et al*. [64] involved daily treatment with a five-grass pollen SLIT tablet outside the pollen season and symptom scoring during two- or four-hour allergen challenges in an allergen challenge facility. In view of these major differences with respect to ‘open-field’ trials, this study was not included in our meta-analysis.

### Meta-analyses

One study [31] did not provide enough information on the dispersion of the data and was not included in our meta-analysis. The results of the meta-analysis for H1-antihistamines as a class are shown in Figure 1. We analyzed 23 treatment arms with a total of 10,206 patients. The overall Hedges' g (95% confidence interval (CI)) was -0.39 (-0.43, -0.35) in a fixed effects model and -0.39 (-0.45, -0.33) in a random effects model.

Figure 2 presents the results of the meta-analysis for nasal corticosteroids, with nine treatment arms totaling 2,549 patients. The overall Hedges' g (95%CI) was –0.55 (-0.63, -0.47) in a fixed effects model and -0.54 (-0.74, -0.34) in a random effects model. The results of the meta-analysis for the leukotriene receptor antagonist montelukast are shown in Figure 3. We analyzed four studies with a total of 2,946 patients. The Hedges' g (95%CI) was –0.23 (-0.30, -0.16) in a fixed effects model and -0.25 (-0.36, -0.14) in a random effects model. The results of the meta-analysis of three trials of an azelastine-fluticasone combination (symptom scores) are shown in Figure 4. The three treatment arms featured a total of 1,703 patients. The overall Hedges' g (95%CI) was –1.00 (-1.10, -0.90) in both fixed effects and random effects models.

Lastly, the results of the meta-analysis for grass pollen SLIT tablets as a class (totaling 3,819 patients in 10 studies) are shown in Figure 5. Hedges' g (95%CI) was -0.30 (-0.36, -0.23) in a fixed effects model and -0.31 (-0.39, -0.22) in a random effects model. We also performed separate analyses for the two different SLIT tablet products. The four studies of five-grass pollen tablets featured a total of 1,612 patients; Hedges' g (95%CI) was -0.40 (-0.50, -0.30) in a fixed effects model and -0.40 (-0.52, -0.29) in a random effects model. The five studies of timothy pollen tablets featured a total of 2,207 patients; Hedges' g (95%CI) was -0.23 (-0.31, -0.16) with both fixed effects and random effects models. A meta-analysis of the combined symptom–medication scores (Additional file 3: Table S3) in grass pollen SLIT trials (excluding the trials by Bufe *et al*. and by Murphy *et al*., for which combined scores were not available [61,63]) led to similar findings (Hedges' g (95%CI): -0.36 (-0.44, -0.28) with both fixed and random effects models). Hence, our meta-analyses confirmed the presence of an effect on symptoms for all drug classes (or for a single drug, for MP29-02 and montelukast), including grass pollen SLIT tablets.

### Relative clinical impact

In order to compare our results for SLIT with those for symptomatic medications, we calculated the RCI (on symptom scores) for each study (Figures 1, 2, 3 and 4). In almost all cases, we and Matricardi *et al*. [23] calculated the same RCI for a given trial. The calculated weighted mean (range) RCIs were -29.6% (-23% to -37%) for five-grass SLIT tablets, -19.2% (-6% to -29%) for timothy SLIT tablets, -15.0% (-3% to -26%) for second-generation H1-antihistamines, -23.5% (-7% to -54%) for nasal corticosteroids, -17.1% (-15% to -20%) for the azelastine-fluticasone combination MP29-02 and -6.5% (-3% to -10%) for montelukast. It should be noted than in head-to-head studies, MP29-02 showed greater efficacy than intranasal fluticasone [57]. Importantly, comparison of these RCIs indicated that the grass SLIT tablets' effect on symptoms (*P* < 0.05) was significantly greater than that of H1 antihistamines and the leukotriene receptor antagonist montelukast and was similar to that of nasal corticosteroids and MP29-02. In the allergen challenge trial by Horak [64], the RCI was -29% (based on mean scores) or -33% (based on median scores). These values are slightly higher than the weighted mean RCIs (based on symptom scores or combined scores) calculated for natural exposure trials of the five-grass pollen SLIT tablets.

The largest RCI (-54%) was obtained for mometasone in a study by Graft *et al*. [50]. This high value may have been due to the atypical study design, since mometasone furoate nasal spray was administered prophylactically for four weeks prior to the expected onset of the ragweed pollen season and then for a further four weeks; efficacy was calculated over the third and fourth weeks of the season.

We also calculated the RCIs for the two types of grass SLIT tablets on the basis of combined symptom and medication scores. There were no marked differences with respect to the RCIs calculated from the symptom score alone (Figure 5 and Additional file 3: Table S3). The weighted mean RCI was -28.8% (instead of -29.6% on the basis of the symptom scores) for the five-grass pollen SLIT tablets and -25.8% (instead of -21.5%) for the timothy pollen SLIT tablets. The overall RCI for grass pollen SLIT tablets as a class (that is, five-grass and timothy) was -28.8% (instead of -23.6%).

## Discussion

### Meta-analyses

Our meta-analyses confirmed the presence of an effect on symptoms for all drug classes (or single drugs, for MP29-02 and montelukast), including grass pollen SLIT tablets. The Hedges' g values calculated here were compatible with those found in the literature data. For example, we found a value of 0.39 for the antihistamines as a class; this may be compared with Compalati *et al*.'s value of 0.42 for fexofenadine [65], Mösges *et al*.'s values of 0.59 for levocetirizine and 0.21 for loratadine [66], and Compalati and Canonica's value of 0.37 for rupatadine [67]. In contrast, Matricardi *et al*. calculated a value of 1.00 for antihistamines as a class; however, the latter meta-analysis included a number of small studies with large effect sizes [23]. The values for montelukast are very consistent: 0.23 in the present study and 0.24 according to both Matricardi *et al*. [23] and Rodrigo *et al*. [68]. Lastly, we calculated a Hedges' g of 0.55 for corticosteroids as a class; Matricardi *et al*.'s value for mometasone was 0.47 [23]. These similarities indicate that our selected studies form a valid basis for further analysis (that is, calculation of the RCI).

### The RCI of SLIT is as large as that of nasal corticosteroids

We studied the degree of symptom relief (relative to placebo) provided by recently approved symptomatic medications and tablet formulations of SLIT products. Despite mechanistic differences in the mechanism of action of these two treatment approaches, the current evidence from the recent, well-powered, stringent, clinical studies analyzed here suggests that grass pollen SLIT tablets provide a greater degree of symptom relief in SAR than certain symptomatic drugs or drug classes (such as the leukotriene receptor antagonist montelukast and second-generation H1-antihistamines) and much the same degree of relief as nasal corticosteroids and an azelastine-fluticasone combination. This finding is especially striking because a number of methodological factors reduce the apparent magnitude of effect in AIT clinical trials. It is problematic to compare the symptomatic medication RCIs calculated in the present study with mean values in the meta-analyses performed and reported by Wilson *et al.* (-18% for nasal corticosteroids, -7% for oral antihistamines and -5% for montelukast [69]) and Benninger *et al.* (-40.7% for nasal corticosteroids and -23.5% for oral antihistamines [70]) because the latter studies used a different calculation method. However, the order of these drug classes in each analysis is consistent (nasal corticosteroids > oral antihistamines > montelukast). It should be borne in mind that within a given symptomatic drug class, members may differ in their efficacy and tolerance. Using a therapeutic index score, Schäfer *et al*. suggested that there were differences between intranasal corticosteroids [71]. Likewise, a meta-analysis from open-label prospective observational studies performed by Mösges *et al*. suggested that levocetirizine is significantly more effective than desloratadine, ebastine and fexofenadine [72]. However, a number of the studies analyzed in the present paper or in the literature made head-to-head comparisons between marketed nasal corticosteroids or between antihistamines. The differences in efficacy were either not significant or were inconsistent from one study to another [33,34,44,50,51].

### Calculation of the RCI

The World Allergy Organization's definition of the RCI (as applied by Matricardi *et al*. [23]) is based solely on the relative mean active treatment versus placebo difference in scores calculated over a defined period (usually the pollen season as a whole for the SLIT studies) [29]. The method is easily applicable to SLIT or SCIT trials lacking a low, pre-season baseline score but its use in short-term pharmacotherapy trials (in which a high, peak-season baseline score is available) can be criticized. Indeed, we excluded a number of pharmacotherapy trials in which the RCI was estimated as the percentage difference between reductions in scores (that is, without reporting the baseline and final scores). In the absence of head-to-head SLIT tablet versus pharmacotherapy trials (which would be difficult to design, implement and interpret), we believe that the RCI affords a meaningful comparison.

### Methodological differences in the clinical assessment of SLIT products versus symptomatic medications

It was only in 2009 that the European Medicines Agency's guideline on the clinical development of AIT products [73] came into force after being released as a draft for consultation in 2007. The recent clinical development of tablet SLIT formulations has closely followed these evidence-based guidelines. However, several methodological factors decrease the apparent RCI for SLIT and, conversely, increase the apparent RCI for symptomatic medications (below and Additional file 4: Table S4).

### Total versus partial symptom scores

The etiological nature of AIT means that clinical trials in this field generally use total symptom scores, in which nasal symptoms, ocular symptoms and (sometimes) other local or individual parameters (coughing, wheezing, ear itch, and so on) are taken into account. In symptomatic medication trials and depending on the drug's pharmacological action, certain symptoms are sometimes excluded from the efficacy scores. The failure to score individual symptoms that are at least partly treatment-refractive (for example, nasal symptoms for antihistamines) may thus prompt overestimation of the RCI for some symptomatic medications.

### Rescue medication use

For ethical reasons, rescue medications cannot be prohibited in month- or year-long SLIT or SCIT trials. The experimental data (that is, medication scores) show that rescue medication use is greater in placebo groups than in active treatment groups. This factor reduces the difference in mean symptom scores between the SLIT and placebo groups and thus leads to underestimation of the RCI for SLIT products. Overall, there were few marked differences between the RCIs calculated from symptom scores and those calculated from combined scores in a given trial (respectively, -27.4% and -30.8% for Didier *et al*. [59], -27.9% and -26.0% for Wahn *et al*. [60], and -18.3% and -20.05% for Nelson *et al*. [16], for example).

### Trial design and duration, patient recruitment, randomization and baseline scores

AIT products and pharmacotherapy products differ markedly in terms of the typical study period in SAR. Symptomatic medication trials typically evaluate symptom relief over a two-week period during the pollen season. In contrast, the efficacy of SLIT (and indeed SCIT) is studied over a whole pollen season (up to two months).

### Disease severity

The mean disease severity in SLIT (and SCIT) trials is usually lower than in symptomatic medication trials, for two main reasons: trial duration and patient recruitment. Firstly, allergen exposure (and thus disease severity) in SLIT trials will fluctuate over the month- or year-long study period, giving peaks and troughs of disease activity. In contrast, symptomatic medications are tested over short periods at or around the pollen peak, when disease severity is high and highly symptomatic patients can be easily recruited. In a SLIT trial, treatment is initiated before the start of the expected pollen season (that is, when patients are asymptomatic). Hence, investigators can never be sure that randomized patients will actually be symptomatic during the coming study. This limitation ‘dilutes’ the level of disease severity. This SLIT versus symptomatic drug difference can be exemplified by estimating the relative SAR severity in the placebo group. In the trials selected in the present meta-analysis, we expressed the mean symptom score as a percentage of the maximum possible symptom score. In the SLIT trials, the mean (range) relative SAR severity score in the placebo group was 24.7% (18% to 34%). In the symptomatic medication trials, the mean (range) relative SAR severity score was 48.7% (30% to 67%) in antihistamine trials, 52.7% (20% to 65%) in nasal corticosteroid trials, 62.2% (58% to 64%) in montelukast trials and 66.5% (64% to 68%) in trials of the azelastine-fluticasone combination.

In SLIT trials, there are several lines of evidence to suggest that greater mean disease severity in patients is associated with a greater RCI (and, conversely, that low mean disease activity in patients reduces the apparent RCI). It is possible to identify high-severity patients within a SLIT trial, so that this subpopulation can then be more fairly compared with high-severity patients in symptomatic medication trials. Firstly, Bufe *et al*.'s study [61] in a pediatric population found RCIs of -24% for the grass pollen season as a whole, -25% for the 15-day peak grass pollen season and -28% for the ‘high-level’ grass pollen season (the period over 30 grains/m^3^). Hence, for SLIT products, low disease activity at the start and end of the pollen season reduces the mean RCI calculated over the season as a whole. Secondly, a novel way to focus on patients in SLIT trials with high disease burdens (thus mirroring experimental conditions in symptomatic medication trials) involves a prespecified, *post hoc* tertile analysis. Howarth *et al.* has applied this approach [74] to three large SLIT clinical trials [19,59,60]. Study centers were grouped into low, middle and high tertiles according to the average RTSS or AAdSS observed in each center's placebo patients. The high-severity tertile (in which the relative severity of SAR was 34%, rather than 27% for the placebo group as a whole) corresponds most closely to the population typically recruited in symptomatic medication trials. After calculating the RCIs on the basis of the average RTSS and the AAdSS for all three studies, Howarth *et al*. found that the greatest RCI was always observed in the high tertile, that is, the centers in which patients were most strongly affected by pollen [74]. When calculated from the AAdSS for the high tertile in four studies of five-grass pollen SLIT tablets, we found that the weighted mean (range) RCI was –37.1% (-26% to -45%). Thirdly, Durham [75] published an analysis of ‘days with severe symptoms’ in clinical trial patients taking a timothy grass tablet. Even though this analysis was based on individual severity scores (rather than groups of centers), Durham *et al*. came to the same conclusion: the more severe the symptoms, the greater the clinical impact of SLIT. Recently, Durham *et al*. [76] have further shown that the size of the treatment effect over five pollen seasons in a long-term trial of timothy SLIT tablets was highly correlated with the cumulative pollen exposure at the start of the season. In particular, the SLIT versus placebo difference in the weighted rhinoconjunctivitis combined symptom and medication score increased as the pollen count increased (reaching about 33% for the highest pollen count).

An interesting question relates to whether the RCI for SLIT products and symptomatic medications changes over time during long-term use (that is, from one year or treatment season to another). The multiseason studies of grass pollen SLIT tablets provide a few indications [18,19]. In the trial by Durham *et al*., the RCIs for the treatment years one, two and three (based on the RTSS) were -0.31, -0.36, and -0.29, respectively [18]. On this basis, Durham *et al*. considered that the ‘reductions in rhinoconjunctivitis symptom and medication scores and the increase in quality of life and percentage symptom- and medication-free days one year after treatment were all similar to the treatment effect at the end of the three-year treatment period’ [18]. Based on the three-season data for the AAdSS in the study by Didier *et al*., the respective RCI for seasons one, two and three were -0.20, -0.34, and -0.37 [19]. However, it must be borne in mind that the mean pollen count (and, thus, the severity of disease) varied from one treatment year or season to the next. This factor is likely to be the major factor involved in the variation of the measured RCI (see below). Due to the absence of a persistent, long-term effect of symptomatic drugs, there is no reason to believe that their efficacy in SAR will change year-on-year.

In summary, an ‘unbiased’ comparison between SLIT and symptomatic medications would have to be performed with the most similar possible levels of pollen exposure and symptom severity. As things stand, one can hypothesize that trials of SLIT (generally performed in patients with mild-to-moderate symptoms) tend to underestimate the RCI for these formulations. Estimation of SLIT's effect size on the basis of the RCI observed for the high disease tertile is far from perfect. However, in the absence of robust, large-scale, head-to-head clinical trials, this tertile is an approximation of the conditions encountered in a symptomatic drug trial.

### Limitations of the RCI approach

Although we restricted our selection to investigations of pollen-induced SAR, the studies of symptomatic medications (notably the H1-antihistamines) were variously performed in spring, summer and fall in patients with SAR induced by tree, grass and/or weed pollens. This is an additional source of heterogeneity. In contrast, the SLIT studies all concerned grass-pollen-induced SAR occurring in late spring/early summer. As mentioned above, SLIT trials and symptomatic medication trials differ in terms of the characteristics of the study population and the scoring systems used. The scores in SLIT trials tend to be averaged over a treatment season, whereas those in symptomatic medications trials tend to be point measurements at the end of a short treatment period. Furthermore, the RCI takes account of differences in scale because the comparison is always made with the placebo group in the same trial. In the term ‘relative clinical impact’, the word ‘relative’ means ‘the clinical impact in the active group relative to the placebo group.’ Hence, the RCI can provide a valid (albeit indirect) comparison between SLIT and symptomatic medications.

## Conclusions

In an indirect comparison (as previously performed for SCIT by Matricardi *et al*. [23]), the administration of grass pollen SLIT tablets was associated with a greater RCI (versus placebo) on symptoms than that provided by second-generation H1-antihistamines and a leukotriene receptor antagonist - medications that clearly ‘work’ in clinical practice and whose efficacy is not called into question. These RCIs were obtained despite the presence of methodological factors that mask the efficacy of SLIT.

## Abbreviations

AAdSS: Average Adjusted Symptom Score; ACS: average combined score; Act: active treatment; AIT: allergen immunotherapy; AR: allergic rhinitis; Beclo: beclomethasone; BID: twice daily; CI: confidence interval, DBPC, double-blind, placebo-controlled; hg: Hedges' g; IR: index of reactivity; Mom: mometasone; Plac: placebo; QD: once daily; RCI: relative clinical impact; RTSS: Rhinoconjunctivitis Total Symptom Score; SAR: seasonal allergic rhinitis; SCIT: subcutaneous allergen immunotherapy; SLIT: sublingual allergen immunotherapy; SQ-T: standardized quality tablet; TCS: total combined score; TnNSS: total nasal symptom score with n individual symptoms; TNSS: total nasal symptom score; TnSS: total symptom score with n individual symptoms; TOSS: total ocular symptom score; TSS: total symptom score; w: weighting; WCS: weighted combined score.

## Competing interests

Philippe Devillier has received consulting fees, honoraria for lectures and/or research funding from Schering-Plough-MSD, Sanofi-Aventis, GlaxoSmithKline, Chiesi, AstraZeneca, ALK, Stallergenes. Jean-François Dreyfus has received consulting fees from Stallergenes. Pascal Demoly is a consultant and a speaker for Stallergenes, ALK and Chiesi and was a speaker for Merck, Astra Zeneca, Menarini and GlaxoSmithKline. Moises A. Calderon has received consulting fees, honoraria for lectures and/or research funding from ALK-Abello, Merck, Stallergenes, Hal Allergy and Allergopharma.

## Authors' contributions

All authors selected studies for analysis. PD and JFD extracted data from publications and calculated the RCIs. JFD performed the meta-analysis. All authors were involved in drafting the manuscript. The authors accept full responsibility for the work and the decision to publish. All authors read and approved the final manuscript.

## Pre-publication history

The pre-publication history for this paper can be accessed here:

http://www.biomedcentral.com/1741-7015/12/71/prepub

## Supplementary Material

Additional file 1: Table S1 List of studies initially considered but not selected, together with the reasons for non-selection. Click here for file

Additional file 2: Table S2 Pharmacotherapy and grass pollen SLIT tablet trials in seasonal allergic rhinitis. T*n*NSS: total nasal symptom score with *n* symptoms; T*n*SS: total symptom score with *n* symptoms; T*n*OSS: total ocular symptom score with *n* symptoms; QD: once daily, BID; twice daily; IR: index of reactivity; SQ-T: standardized quality tablet. RTSS: rhinoconjunctivitis total symptom score; ^**‡**^as used in the meta-analysis; *as used in the meta-analysis and generally (but not always) the study's stated primary efficacy criterion. Click here for file

Additional file 3: Table S3 RCI and meta-analysis of efficacy (based on combined scores) for grass pollen SLIT tablets. N Act: number of subjects in the active treatment group; Mean Act: mean score in the active treatment group; SD Act; standard deviation for the score in the active treatment group; N Plac: number of subjects in the placebo group; Mean Plac: mean score in the placebo group; SD Plac: standard deviation for the score in the placebo group; RCI: relative clinical impact; hg: Hedges' g; ci-: lower confidence interval; ci+: upper confidence interval; z: z score: p: *P*-value; w: weighting; ACS: average combined score; TCS: total combined score = daily symptom score + daily medication score; WCS: weighted combined score = (daily symptom score/maximum symptom score))/(1- daily medication score - maximum symptom score). Click here for file

Additional file 4: Table S4 Methodological differences in the evaluation of treatments for pollen-induced seasonal allergic rhinitis. T6SS: total symptom score with six individual symptoms; T4SS: total symptom score with four individual symptoms. Click here for file
