# Supplementary material for: A meta-analysis of sublingual allergen immunotherapy and pharmacotherapy in pollen-induced seasonal allergic rhinoconjunctivitis
Source: BMC Med. 2014 May 1;12:71. doi: 10.1186/1741-7015-12-71 (PMC4101870; doi:10.1186/1741-7015-12-71)
Supplement: Additional file 1: Table S1 — List of studies initially considered but not selected, together with the reasons for non-selection. [file 1741-7015-12-71-S1.pdf]

Additional Table 1. List of studies initially considered but not selected , together with the reasons for non-selection.

Study	Medication	Reason for non-selection
Bronsky AAAI 1997	mometasone furoate	<100 participants in the smallest arm
Meltzer JACI 1998	mometasone furoate	<100 participants in the smallest arm
Berkowitz Allergy 1999	mometasone furoate	<100 participants in the smallest arm
Meltzer JACI 2000	montelukast	<100 participants in the smallest arm
Pullerits JACI 2002	montelukast	<100 participants in the smallest arm
Wilson Clin Exp Allergy 2002	desloratadine	<100 participants in the smallest arm
Stuck Allergy 2003	mometasone furoate	<100 participants in the smallest arm
Ciprandi Clin Exp Allergy 2004	desloratadine	<100 participants in the smallest arm
Cyr IAAI 2005	desloratadine	<100 participants in the smallest arm
Bronsky Allergy Asthma Proc 1998	fexofenadine	Baseline and/or post-treatment scores are not given - only reductions in score are quoted. Not possible to calculate the RCI
Casale Allergy Asthma Proc 1999	fexofenadine	Reduction in score only shown graphically. Not possible to calculate the RCI
Meltzer JACI 1999	mometasone furoate	Baseline and/or post-treatment scores are not given - only reductions in score are quoted. Not possible to calculate the RCI
Casale Allergy Asthma Proc 1999	fexofenadine	Reduction in score only shown graphically. Not possible to calculate the RCI
Van Cauwenberge Clin Exp Allergy 2000	fexofenadine	Baseline and/or post-treatment scores are not given - only reductions in score are quoted. Not possible to calculate the RCI
Van Cauwenberge Clin Exp Allergy 2000	loratidine	Baseline and/or post-treatment scores are not given - only reductions in score are quoted. Not possible to calculate the RCI
Salmun BMC Family Practice 2002	desloratadine	Baseline and/or post-treatment scores are not given - only reductions in score are given (as a Figure). Not possible to calculate the RCI
Gawchik AAAI 2003	mometasone furoate	Nightime score only
Berger AAAI 2003	desloratadine + placebo spray	Not desloratadine alone (desloratadine 5 mg in capsules plus placebo saline nasal spray)
Ratner IAAI 2004	loratadine	Not an individual study - meta-analysis of four randomized, double-blind, placebo-controlled, parallelgroup clinical trials

Ratner IAAI 2004	ebastine	Not an individual study - meta-analysis of four randomized, double-blind, placebo-controlled, parallelgroup clinical trials
Meltzer AAAI. 2005	olopatadine	Baseline and/or post-treatment scores are not given - only reductions in score are quoted. Not possible to calculate the RCI
Ratner AAAI 2005	olopatadine	Baseline and/or post-treatment scores are not given - only reductions in score are quoted. Not possible to calculate the RCI
Meltzer AAAI. 2005	olopatadine	Baseline and/or post-treatment scores are not given - only reductions in score are quoted. Not possible to calculate the RCI
Berger Allergy Asthma Proc 2006	fexofenadine	Only reductions in score are given (as a Figure). Not possible to calculate the RCI
Berger Allergy Asthma Proc 2006	desloratadine	Only reductions in score are given (as a Figure). Not possible to calculate the RCI
Berger AAAI 2006	azelastine	No placebo group
Berger AAAI 2006	cetirizine	No placebo group
Meltzer Ann Allergy Immunol 2006	desloratadine	Only reductions in score are given (as a Figure). Not possible to calculate the RCI
Anolik IAAI 2008	mometasone furoate	Ocular symptoms only
Grubbe JIACI 2009	desloratadine	Combination treatments only : (i) desloratadine+pseudoephedrine, (ii) desloratadine+placebo tablet and (iii) pseudoephedrine.
Demoly AAAI 2009	desloratadine	Only reductions in score are given (as a Figure). Not possible to calculate the RCI

REFERENCES

- Bronsky EA, Aaronson DW, Berkowitz RB, Chervinsky P, Graft D, Kaiser HB, Moss B, Nathan RA, Pearlman DS, Ratner PH, Adelglass JM, Southern DL, van Bavel J, Hampel F, Stricker WE, Fourré JA, Cuss FM, Nolop KB: **Dose ranging study of mometasone furoate (Nasonex) in seasonal allergic rhinitis.** *Ann Allergy Asthma Immunol* 1997, **79:**51-6.

- Meltzer EO, Jalowayski AA, Orgel HA, Harris AG: **Subjective and objective assessments in patients with seasonal allergic rhinitis: effects of therapy with mometasone furoate nasal spray.** *J Allergy Clin Immunol* 1998, **102:**39-49.
- Berkowitz RB, Bernstein DI, LaForce C, Pedinoff AJ, Rooklin AR, Damaraju CR, Mesarina-Wicki B, Nolop KB: **Onset of action of mometasone furoate nasal spray (NASONEX) in seasonal allergic rhinitis.** *Allergy* 1999, **54:**64-9.
- Meltzer EO, Malmstrom K, Lu S, Prenner BM, Wei LX, Weinstein SF, Wolfe JD, Reiss TF: **Concomitant montelukast and loratadine as treatment for seasonal allergic rhinitis: a randomized, placebo-controlled clinical trial.** *J Allergy Clin Immunol* 2000, **105:**917-22.
- Pullerits T, Praks L, Ristioja V, Lötvall J. **Comparison of a nasal glucocorticoid, antileukotriene, and a combination of antileukotriene and antihistamine in the treatment of seasonal allergic rhinitis**. *J Allergy Clin Immunol*. 2002, **109**:949-55.
- Wilson AM, Haggart K, Sims EJ, Lipworth BJ. **Effects of fexofenadine and desloratadine on subjective and objective measures of nasal congestion in seasonal allergic rhinitis**. *Clin Exp Allergy*. 2002;**32**:1504-9.
- Stuck BA, Blum A, Hagner AE, Hummel T, Klimek L, Hörmann K: **Mometasone furoate nasal spray improves olfactory performance in seasonal allergic rhinitis.** *Allergy* 2003, **58:**1195.
- Ciprandi G, Cirillo I, Vizzaccaro A, Tosca MA: **Levocetirizine improves nasal obstruction and modulates cytokine pattern in patients with seasonal allergic rhinitis: a pilot study.** *Clin Exp Allergy* 2004, **34:**958-64.

- Cyr MM, Hayes LM, Crawford L, Baatjes AJ, Keith PK, Denburg JA: **The effect of desloratadine on eosinophil/basophil progenitors and other inflammatory markers in seasonal allergic rhinitis: a placebo-controlled randomized study.** *Int Arch Allergy Immunol* 2005 **138:**209-16.
- Bronsky EA, Falliers CJ, Kaiser HB, Ahlbrandt R, Mason JM: **Effectiveness and safety of fexofenadine, a new nonsedating H1-receptor antagonist, in the treatment of fall allergies.** *Allergy Asthma Proc* 1998, **19:**135-41.
- Casale TB, Andrade C, Qu R: **Safety and efficacy of once-daily fexofenadine HCl in the treatment of autumn seasonal allergic rhinitis.** *Allergy Asthma Proc* 1999, **20:**193-8.
- Meltzer EO, Berger WE, Berkowitz RB, Bronsky EA, Dvorin DJ, Finn AF, Galant SP, Grossman J, Hampel FC, Ratner PH, Ruff ME, Schenkel EJ, Segal AT, Segall N, Stewart GE 2nd, Tripathy I, Skoner DP, Anolik R, Dockhorn RJ, van Bavel J, Mesarina-Wicki B, Nolop K: **A dose-ranging study of mometasone furoate aqueous nasal spray in children with seasonal allergic rhinitis**. *J Allergy Clin Immunol* 1999, **104:**107-14.
- Van Cauwenberge P, Juniper EF: **Comparison of the efficacy, safety and quality of life provided by fexofenadine hydrochloride 120 mg, loratadine 10 mg and placebo administered once daily for the treatment of seasonal allergic rhinitis.** *Clin Exp Allergy* 2000, **30:**891-9.
- Salmun LM, Lorber R: **24-hour efficacy of once-daily desloratadine therapy in patients with seasonal allergic rhinitis [ISRCTN32042139].** *BMC Fam Pract* 2002, 3:14.

- Gawchik S, Goldstein S, Prenner B, John A: **Relief of cough and nasal symptoms associated with allergic rhinitis by mometasone furoate nasal spray.** *Ann Allergy Asthma Immunol* 2003, **90:**416-21.
- Berger WE, White MV; Rhinitis Study Group: **Efficacy of azelastine nasal spray in patients with an unsatisfactory response to loratadine.** *Ann Allergy Asthma Immunol* 2003, **91:**205-11.
- Ratner P, Hampel F Jr, Van Bavel J, Howland W 3rd: **Efficacy and safety of ebastine 20 mg compared to loratadine 10 mg once daily in the treatment of seasonal allergic rhinitis: a randomized, double-blind, placebo-controlled study.** *Int Arch Allergy Immunol* 2004, **133:**371-9.
- Meltzer EO, Hampel FC, Ratner PH, Bernstein DI, Larsen LV, Berger WE, Finn AF Jr, Marple BF, Roland PS, Wall GM, Brubaker MJ, Dimas C, Potts SL, Silver LH, Barnes JR: **Safety and efficacy of olopatadine hydrochloride nasal spray for the treatment of seasonal allergic rhinitis.** *Ann Allergy Asthma Immunol* 2005, **95:**600-6.
- Ratner PH, Hampel FC, Amar NJ, van Bavel JH, Mohar D, Marple BF, Roland PS, Wall GM, Brubaker MJ, Drake M, Turner D, Silver LH: **Safety and efficacy of olopatadine hydrochloride nasal spray for the treatment of seasonal allergic rhinitis to mountain cedar.** *Ann Allergy Asthma Immunol* 2005, **95:**474-9.
- Berger W, Hampel F Jr, Bernstein J, Shah S, Sacks H, Meltzer EO: **Impact of azelastine nasal spray on symptoms and quality of life compared with cetirizine oral tablets in patients with seasonal allergic rhinitis.** *Ann Allergy Asthma Immunol* 2006 **97:**375-81.

- Meltzer EO, Jalowayski AA, Vogt K, Iezzoni D, Harris AG: **Effect of desloratadine therapy on symptom scores and measures of nasal patency in seasonal allergic rhinitis: results of a single-center, placebo-controlled trial.** *Ann Allergy Asthma Immunol* 2006, **96:**363-8.
- Anolik R, Nathan RA, Schenkel E, Danzig MR, Gates D, Varghese S: **Intranasal mometasone furoate alleviates the ocular symptoms associated with seasonal allergic rhinitis: results of a post hoc analysis.** *Int Arch Allergy Immunol* 2008, **147:**323-30.
- Grubbe RE, Lumry WR, Anolik R: **Efficacy and safety of desloratadine/pseudoephedrine combination vs its components in seasonal allergic rhinitis.** *J Investig Allergol Clin Immunol* 2009, **19:**117-24.
- Demoly P, Dreyfus I, Dhivert-Donnadieu H, Mesbah K: **Desloratadine for the treatment of cypress pollen-induced allergic rhinitis.** *Ann Allergy Asthma Immunol* 2009 **103:**260-6.
