# Supplementary material for: A meta-analysis of sublingual allergen immunotherapy and pharmacotherapy in pollen-induced seasonal allergic rhinoconjunctivitis
Source: BMC Med. 2014 May 1;12:71. doi: 10.1186/1741-7015-12-71 (PMC4101870; doi:10.1186/1741-7015-12-71)
Supplement: Additional file 2: Table S2 — Pharmacotherapy and grass pollen SLIT tablet trials in seasonal allergic rhinitis. TnNSS: total nasal symptom score with n symptoms; TnSS: total symptom score with n symptoms; TnOSS: total ocular symptom score with n symptoms; QD: once daily, BID; twice daily; IR: index of reactivity; SQ-T: standardized quality tablet. RTSS: rhinoconjunctivitis total symptom score; ‡as used in the meta-analysis; *as used in the meta-analysis and generally (but not always) the study's stated primary efficacy criterion. [file 1741-7015-12-71-S2.pdf]

Additional Table 2. Pharmacotherapy and grass pollen SLIT tablet trials in seasonal allergic rhinitis

H1-antihistamines							
**Product**	**Dose**	**Publication**	**Population age**	**n (Active)‡**	**n (Placebo)‡**	**Type of score***	**Treatment period**
desloratadine	5 mg	Meltzer Clin Drug Invest 2001	12 to 65, no mean or median given	172	174	Spring, T8SS, reflective 12-hr am/pm	14 days
desloratadine	5 mg	Meltzer Clin Drug Invest 2001	12 to 65, no mean or median given	164	164	Fall, T8SS, reflective 12-hr am/pm	14 days
desloratadine	5 mg	Raphael AAAI 2006	12 to 65 (mean Ac 34.0, Pl 36.4)	198	197	T8SS, daily 24-hour reflective	7 days
desloratadine	5 mg	Pradalier Allergy 2007	18 or older (mean Ac 32.7, Pl 32.4)	234	249	T8SS, am/pm	14 days
desloratadine	5 mg	Bachert Allergy 2009	12 to 70 (mean Ac	242	245	T8SS, daily	14

			29.8, Pl 30.6				
bilastine	20 mg	Bachert Allergy 2009	12 to 70 (mean Ac 30.6, Pl 30.6)	233	245	T10SS, 12-hr reflective	14 days
bilastine	20 mg	Kuna CEA 2009	12 to 70 (mean Ac 31.16; Pl 30.2)	216	205	reflective T7SS	14 days
levocetirizine	5 mg	Leynadier Acta ORL Belg 2001	18 to 72 [median Ac 31, Pl 31)	115	118	T4SS, 24-hour reflective	14 days
levocetirizine	5 mg	Segall AAAI 2010	18 to 65 (mean Ac 38.7, Pl 37.5)	284	290	T5SS, 24-hour reflective	14 days
loratadine	10 mg	Nayak AAAI 2002	15 to 82 (mean Ac 37, Pl 37)	301	149	T4NSS daytime	14 days
loratadine	10 mg	Philip Clin Exp Allergy 2002	15 to 81 (mean Ac 36, Pl 36)	602	352	T8SS daytime	14 days
loratadine	10 mg	van Adelsberg AAAI 2003	15 to 85 (mean Ac 35, Pl 36)	171	521	T4NSS, daytime	14 days
loratadine	10 mg	van Adelsberg Allergy 2003	15 to 82 (mean Ac 39, Pl 36)	170	410	T4NSS	14 days

loratadine	10 mg	Anolik AAAI 2008	12 to 66 (mean Ac 25, Pl 26)	173	157	T8SS, patient-reported	15 days
fexofenadine	120 mg	Bernstein AAAI 1997	12 to 65 (mean Ac 32; Pl 33)	144	141	T8SS, 12-hr reflec.	14 days
fexofenadine	120 mg	Howarth JACI 1999	12 to 66 (mean Ac 33 ; Pl 34)	211	201	T4SS, 24-hr reflec.TSS	14 days
fexofenadine	30 mg	Wahn JACI 2003	6 to 11 (mean Ac 8.8, Pl 8.8)	460	465	T9SS, PM-reflective	14 days
cetirizine	10 mg	Kuna CEA 2009	12 to 70 (mean Ac 31.8; Pl 30.2)	225	205	reflective T7SS	14 days
cetirizine	10 mg	Howarth JACI 1999	12 to 66 (mean Ac 33; Pl 34)	207	201	T4SS, 24-hr reflec.TSS	14 days
azelastine	2 sprays per nostril twice daily daily	Berger AAAI 2003	12 to 79 (mean Ac 36; Pl 37)	108	111	T4NSS, 12-hour reflective	14 days
azelastine	1 spray per	LaForce AAAI 2004	12 to 80 (mean Ac 35;	112	110	T4NSS, 12-hour	14 days

	nostril, twice daily		Pl 35)			reflective	
azelastine	1 spray per nostril, twice daily	Lumry AAAI 2007 (study 1)	12 to 73 (mean Ac 33; Pl 35)	139	141	T4NSS, combined morning + evening reflective	14 days
azelastine	1 spray per nostril, twice daily	Lumry AAAI 2007 (study 2)	12 to 75 (mean Ac 35; Pl 36)	137	137	T4NSS, combined morning + evening reflective	14 days
Nasal corticoids							
**Product**	**Dose**	**Publication**	**Population age**	**n (Active)‡**	**n (Placebo)‡**	**Type of score***	**Treatment period**
ciclesonide	200 µg QD	Ratner JACI 2006	12 or older (mean Ac 39, Pl 41)	164	163	T4NSS, 12-hour reflective	14 days
beclomethasone	168 µg BID	Graft JACI 1996	12 to 69 (mean Ac 34,	112	104	T4NSS	28 days

dipropionate			Pl 34)				
mometasone furoate	200 µg QD	Graft JACI 1996	12 to 69 (mean Ac 36, Pl 34)	114	101	T4NSS	28 days
mometasone furoate	200 µg QD	Hebert Allergy 1996	18 to 73 (mean Ac 33, Pl 33)	122	110	T4NSS (physician-rated)	8 days
mometasone furoate	200 µg QD	Gawchik AAAI 2003	12 or older (mean Ac 34.7, Pl 34.2)	122	123	T4NSS + cough day	14 days
fluticasone furoate	110 µg	Martin Allergy Asthma 2007	12 or older ("mean 39.3", no separate data for Ac and Pl)	127	128	T4NSS, 12-hour reflective	14 days
fluticasone furoate	110 µg	Kaiser JACI 2007	12 or older (mean Ac 35.4, Pl 34.5)	151	148	T4NSS, 12-hour reflective	14 days
mometasone furoate	200 µg QD	Prenner JACI 2010	12 or older (mean Ac 34.5, Pl 36.8)	220	209	T4NSS, am + pm reflective	15 days
mometasone furoate	200 µg QD	Anolik AAAI 2008	12 to 71 (mean Ac 26, Pl 26)	166	165	T8SS, patient-reported	15 days

Montelukast							
**Product**	**Dose**	**Publication**	**Population age**	**n (Active)‡**	**n (Placebo)‡**	**Type of score***	**Treatment period**
montelukast	10 mg	van Adelsberg Allergy 2003	15 to 82 (mean Ac 36, Pl 36)	448	451	T4NSS	14 days
montelukast	10 mg	van Adelsberg AAAI 2003	15 to 85 (mean Ac 36, Pl 36)	522	521	T4NSS, daytime	14 days
montelukast	10 mg	Philip Clin Exp Allergy 2002	15 to 81 (mean Ac 37, Pl 36)	348	352	T8SS daytime	14 days
montelukast	10 mg	Nayak AAAI 2002	15 to 82 (mean Ac 35, Pl 37)	155	149	T4NSS daytime	14 days
MP29-02 (an azelastine-fluticasone combination)							
**Product**	**Dose**	**Publication**	**Population age**	**n (Active)‡**	**n (Placebo)‡**	**Type of score***	**Treatment period**
MP29-02	137 mg	Carr JACI 2012 (study	>12 (mean Ac , Pl)	207	209	T4NSS, T3OSS:	14 days

	azelastine/50 mg fluticasone propionate	MP4002)				(12-hour reflective and instantaneous)	
MP29-02	137 mg azelastine/50 mg fluticasone propionate	Carr JACI 2012 (study MP4004)	>12 (mean Ac , Pl)	193	200	T4NSS, T3OSS: (12-hour reflective and instantaneous)	14 days
MP29-02	137 mg azelastine/50 mg fluticasone propionate	Carr JACI 2012 (study MP4006)	>12 (mean Ac , Pl)	446	448	T4NSS, T3OSS: (12-hour reflective and instantaneous)	14 days
Grass pollen sublingual allergen immunotherapy tablets							

**Product**	**Dose**	**Publication**	**Population age**	**n (Active)‡**	**n (Placebo)‡**	**Type of score***	**Treatment period**
Five-grass tablet	300 IR	Wahn JACI 2009	5 to 17 (mean Ac 10.5, Pl 11.2)	131	135	T6SS=RTSS	6 months, single treatment season
Five-grass tablet	300 IR	Didier JACI 2007	18 to 45 (mean Ac 28.7, Pl 29.1)	136	148	T6SS=RTSS	6 months, single treatment season
Five-grass tablet	300 IR	Cox JACI 2012	18 to 65 (mean Ac 36.8, Pl 37.6)	208	228	T6SS=RTSS	6 months, single treatment season
Five-grass tablet	300 IR(group with 2 months of	Didier JACI 2011	18 to 50 (mean Ac 30.4, Pl 30.2)	147	165	T6SS=RTSS	3.5 months each year for 3 years;

	pre-seasonal treatment)						efficacy data analyzed for the last season
Five-grass tablet	300 IR (group with 4 months of pre-seasonal treatment)	Didier JACI 2011	18 to 50 (mean Ac 30.9, Pl 30.2)	149	165	T6SS=RTSS	5.5 months each year for 3 years; efficacy data analyzed for the last season
Timothy tablet	75,000 SQ-T	Nelson JACI 2011	18 to 65 (mean Ac 35,9 , Pl 35,9)	208	225	T6SS=RTSS	6 months, single treatment season
Timothy tablet	75,000 SQ-T	Blaiss JACI 2011	5 to 18 (mean Ac 12.1, Pl 12.6)	173	167	T6SS=RTSS	6 months, single

							treatment season
Timothy tablet	75,000 SQ-T	Bufe JACI 2009	5 to 16 (mean Ac 10.1, Pl 10.1)	117	121	T6SS=RTSS	6 months, single treatment season
Timothy tablet	75,000 SQ-T	Durham JACI 2010	18 to 65 (means not reported)	160	127	T6SS=RTSS	3 years, efficacy data analyzed for the last season
Timothy tablet	75,000 SQ-T	Durham JACI 2006	18 to 65 (mean Ac 36, Pl 36)	294	286	T6SS=RTSS	4 months, single treatment season
Timothy tablet	75,000 SQ-T	Murphy J Negat Results Biomed. 2013	18 to 65 (mean Ac 35.9, Pl 35.9)	163	166	T6SS=RTSS	3 to 5 months,

							single treatment season

T*n*NSS: total nasal symptom score with *n* symptoms; T*n*SS: total symptom score with *n* symptoms; T*n*OSS: total ocular symptom score with *n* symptoms; QD: once daily, BID; twice daily; IR: index of reactivity; SQ-T: standardized quality tablet. RTSS: rhinoconjunctivitis total symptom score; ‡ as used in the meta-analysis**;** * as used in the meta-analysis and generally (but not always) the study's stated primary efficacy criterion.
