# Supplementary figures and images for: A meta-analysis of sublingual allergen immunotherapy and pharmacotherapy in pollen-induced seasonal allergic rhinoconjunctivitis
Source: BMC Med. 2014 May 1;12:71. doi: 10.1186/1741-7015-12-71 (PMC4101870; doi:10.1186/1741-7015-12-71)

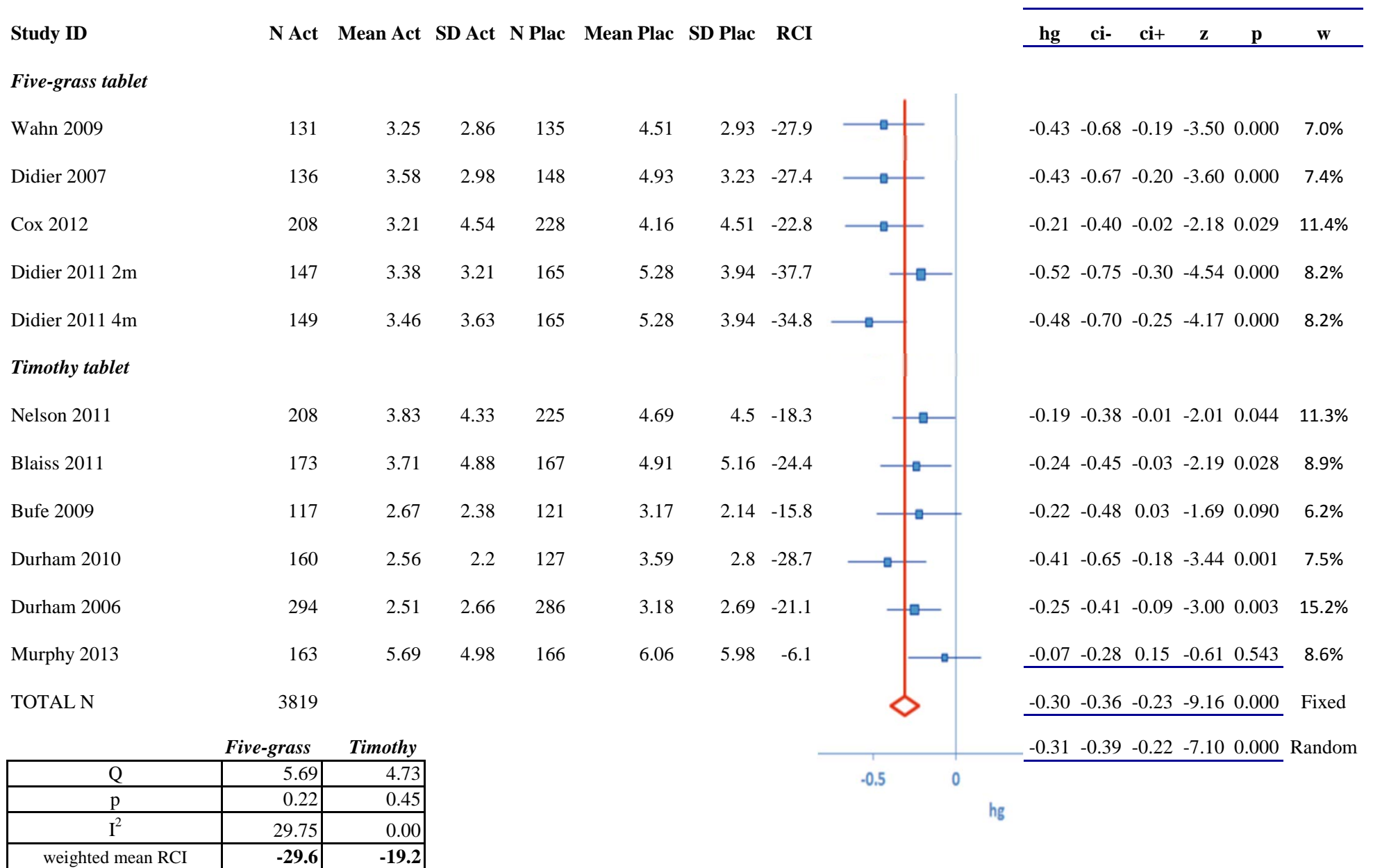

Supplement: Additional file 3: Table S3 — RCI and meta-analysis of efficacy (based on combined scores) for grass pollen SLIT tablets. N Act: number of subjects in the active treatment group; Mean Act: mean score in the active treatment group; SD Act; standard deviation for the score in the active treatment group; N Plac: number of subjects in the placebo group; Mean Plac: mean score in the placebo group; SD Plac: standard deviation for the score in the placebo group; RCI: relative clinical impact; hg: Hedges' g; ci-: lower confidence interval; ci+: upper confidence interval; z: z score: p: P-value; w: weighting; ACS: average combined score; TCS: total combined score = daily symptom score + daily medication score; WCS: weighted combined score = (daily symptom score/maximum symptom score))/(1- daily medication score - maximum symptom score). [file 1741-7015-12-71-S3.pdf]
