# Supplementary material for: A meta-analysis of sublingual allergen immunotherapy and pharmacotherapy in pollen-induced seasonal allergic rhinoconjunctivitis
Source: BMC Med. 2014 May 1;12:71. doi: 10.1186/1741-7015-12-71 (PMC4101870; doi:10.1186/1741-7015-12-71)
Supplement: Additional file 4: Table S4 — Methodological differences in the evaluation of treatments for pollen-induced seasonal allergic rhinitis. T6SS: total symptom score with six individual symptoms; T4SS: total symptom score with four individual symptoms. [file 1741-7015-12-71-S4.pdf]

Additional Table 4. Methodological differences in the evaluation of treatments for pollen-induced seasonal allergic rhinitis

SOURCE OF VARIABILITY	ALLERGEN IMMUNOTHERAPY	SYMPTOMATIC MEDICATIONS	COMMENTS
Study length and design	- Increased variability due to a longer evaluation period (30 days)<br>- Pollen season variability<br>- Use of rescue medication	- Shorter evaluation period (2 weeks)<br>- Less variability in pollen counts<br>- No use of rescue medication	
Primary evaluation criterion	The difference between the mean active treatment (Act) and placebo (Plc) scores over the entire pollen season<br>(30 days, on average)	The relative Act vs. Plc difference in reduction in (high) baseline scores	The evaluation periods differ and penalize allergen immunotherapy (AIT), with dilution of the apparent RCI over the whole season.
Date and method of randomization	The patient is randomized according to his/her disease history (treatment is initiated preseasonally, before the disease symptoms appear)	Randomized patients must exceed a high, minimum symptom score observed at inclusion during the season (i.e. high disease activity is necessarily present).	Inclusion on the basis of disease history alone can lead to memory bias. The likelihood of a treatment effect (improvement in symptoms) in an AIT trial does not solely depend on fluctuations in disease activity
Exposure to allergen	The likelihood of a treatment effect (non-aggravation of symptoms)	The likelihood of the treatment effect	

	depends on the patients' exposure to pollen and the predicted risk of disease occurrence	(improvement of symptoms) depends on fluctuations in disease activity only	
Baseline scores	No baseline (BL) because patients are generally asymptomatic on inclusion (pre-season).	Measurement of a baseline on inclusion yields pre- and post-treatment scores: a relative improvement in symptoms can be measured:<br>([Change in Act from BL/Change in Plc from BL] – 1) * 100	The unpredictability and variability of exposure to the allergen - particularly for pollen - decreases the value of data collected at baseline
Symptom scores	T6SS (4 nasal + 2 ocular) or more	Often a T4SS for H1-antihistamines and a T4NSS for nasal corticosteroids	H1-antihistamine may not take account of nasal congestion.<br>Nasal corticosteroids do not often take ocular symptoms into account
Rescue medication	Allowed, with interindividual variability in use	Prohibited	Represents an additional factor that decreases the RCI for active AIT, since rescue medication use is greater in the Plc group
